# Unique trophoblast chromatin environment mediated by the PcG protein SFMBT2

**DOI:** 10.1242/bio.043638

**Published:** 2019-08-07

**Authors:** Priscilla Tang, Kamelia Miri, Susannah Varmuza

**Affiliations:** Department of Cell and Systems Biology, University of Toronto, 25 Harbord St., Toronto, Ontario M5S 3G5, Canada

**Keywords:** SFMBT2, Trophoblast, ChIP-seq, Placenta, Polycomb

## Abstract

Stem/progenitor cells are maintained by a chromatin environment, mediated in part by Polycomb group (PcG) proteins, which depress differentiation. The trophoblast-specific PcG protein SFMBT2 is known to be required for maintenance of trophoblast progenitors. Rather than binding to trophoblast-specific genes repressed in TSC, SFMBT2 is concentrated at chromocentres and regions rich in repetitive elements, specifically LINE sequences and major satellites, suggesting that it is involved in higher-order organization of the trophoblast genome. It is also found enriched at a subset of ncRNAs. Comparison of ChIP-seq datasets for other chromatin proteins reveals several stereotypical distribution patterns, suggesting that SFMBT2 interacts with several different types of chromatin complexes specific to the trophoblast lineage.

## Abstract (precis)

**Summary:** The PcG protein SFMBT2, essential for placenta development, is distributed mainly to repetitive elements in trophoblast stem cells, arguing for an architectural role in stem cell maintenance.

## INTRODUCTION

Epigenetic biochemistry is as old as cells themselves. It has had billions of years to diversify, which is why it is complex and as evolutionarily varied as it is conserved (Willbanks et al., 2016; Pikaard and Mittelsten Scheid, 2014; Skinner, 2011). Our understanding of the role played by epigenetics in life, the universe and everything, by comparison, is rudimentary, although the development of new tools has accelerated investigation beyond the blunt hammer of DNA methylation.

Pioneering genetic studies in fruit flies have revealed that gene silencing is rooted in complexes of chromatin proteins (Lewis, 1978; Struhl, 1981; Jürgens, 1985). One class that has received particular attention is the PcG of proteins, named after one of the first identified gene silencers, Polycomb, required to maintain posterior spatial expression of homeotic genes in fruit fly larvae (Lewis, 1949, 1978; Jürgens, 1985). The characteristic phenotype – heterozygous ectopic development of sex comb bristles and homozygous lethality due to ectopic expression of homeotic genes – is common to a particular class of proteins that constitute subunits of multi-protein Polycomb Repressive Complexes (PRC1 and PRC2), in addition to other complexes such as PrDUB (Polycomb repressive complex de-ubiquitinase) and PHO-RC (Pleohomeotic repressive complex) (Chittock et al., 2017; Schwartz and Pirrotta, 2013; Golbabapour et al., 2013). In vertebrates, multiple flavours of PRCs have evolved in part as a result of two whole-genome duplication events (Dehal and Boore, 2005; Senthilkumar and Mishra, 2009). This greatly expands the possibilities with respect to developmental innovation.

One such innovation is the placenta. Extraembryonic membranes in egg-laying vertebrates evolved into complex structures in mammals that provide an interface between the developing embryo and the mother, a necessity dictated by the exceedingly small mammalian zygote; there is not enough mass in a mammalian egg to support development of an entity that can survive and feed. Mammalian development therefore takes place in a specialized extraembryonic environment. While there is growing epidemiological evidence that insults during fetal life can have lasting effects on health (Langley-Evans et al., 1999; Sasaki et al., 2014; Nakayama, 2017), there is surprisingly modest interest in examining the role played by the placenta in shaping the fetal developmental environment (Perez-Garcia et al., 2018; Price et al., 2016; Zhang et al., 2015; Suter et al., 2011; Banister et al., 2011).

One area that could benefit from more focused study is the epigenome of the placenta. The ENCODE project at present is greatly enriched by numerous studies of either embryonic stem cells or neural stem cells (Mouse ENCODE Consortium et al., 2012). Very little work has been done to date on the cells that form the placenta. The study we describe in this paper aims to fill some of that deficit.

Placentas are formed from specialized cells called trophoblast (Rossant and Cross, 2001; Cross et al., 2003; Cross, 1998). In mouse embryos, trophoblast cells are one of three early lineages to form before the embryo implants in the mother's uterine wall, the other two lineages represented by embryonic cells and primitive endoderm cells (Cross, 1998; Cross et al., 1994). In mice, these three types of cell can be induced to form specialized stem or progenitor cells *in vitro* – trophoblast stem cells (TSC), embryonic stem cells (ESC) and extraembryonic stem cells (XEN) (Hemberger et al., 2004; Bibel et al., 2004; Kruithof-de Julio et al., 2011). One kind of genetic analysis that has helped define the molecular underpinnings of the trophoblast lineage is the ability of mutant embryos to make TSC. Our lab recently reported that the PcG gene *Sfmbt2* is required for the establishment of TSC, indicating that it may form part of the epigenetic framework that supports trophoblast, and by extension development of the placenta (Miri et al., 2013). *Sfmbt2* was designated a PcG gene because the fruit fly orthologue, *dSfmbt*, displays a classic Polycomb phenotype when mutated (Klymenko et al., 2006). Mammalian SFMBT2 can therefore be reasonably expected to form a complex with other chromatin proteins, and to associate with the genome.

ENCODE datasets comprise, largely, whole-genome sequencing analyses of the occupation of sites by a variety of chromatin proteins (ChIP-seq) and chromosome conformation capture (Hi-C) (ENCODE Project Consortium et al., 2007; ENCODE Project Consortium et al., 2012; Mouse ENCODE Consortium et al., 2012; Dixon et al., 2012). These have allowed investigators to develop novel hypotheses about genome function at a larger scale than was possible even 10 years ago (e.g. topologically associating domains, or TADs, and distal enhancers defined by transcription factor occupancy in specific cell types) (Pope et al., 2014; ENCODE Project Consortium et al., 2012; Zacher et al., 2017). Trophoblast cells are just beginning to acquire epigenomic descriptors. In our study, we analyze the occupancy of the genome in TSC by SFMBT2, a protein known from our genetic studies to be required for the maintenance of the stem cell pool. ChIP-seq analyses have revealed that SFMBT2 may play a unique role in the architecture of TSC, mainly through binding to specific repetitive elements, and association with pericentromeric heterochromatin.

## RESULTS

### Loss of SFMBT2 results in upregulation of genes

**Fig. 1.**
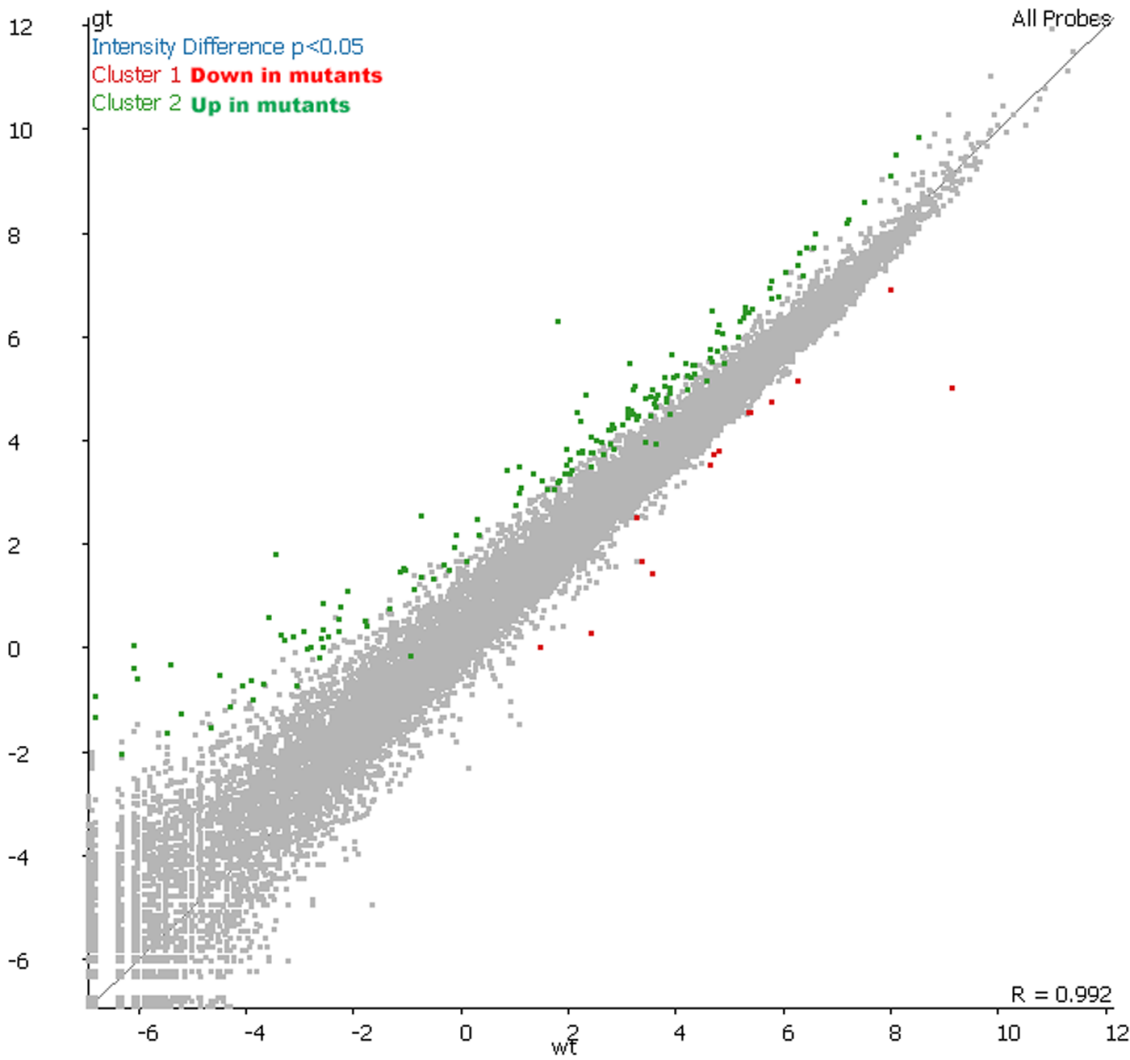
**RNA-seq analysis of *Sfmbt2* null extraembryonic tissues from E7.5 embryos.** Approximately 70% of significantly differentially expressed genes identified in *Sfmbt2gt/gt* extraembryonic tissues were upregulated (green) compared to wild type (wt; *P*<0.05). Few significantly downregulated genes (red) were identified in these tissues. Expression levels of each gene in genetrap mutants (*Sfmbt2gt/gt*) were mapped relative to their wild-type counterparts.

The mammalian SFMBT2 protein is orthologous with the fruit fly dSfmbt, which has been characterized as a Polycomb Group (PcG) protein because the null phenotype of mutants is classic Polycomb (Klymenko et al., 2006; Alfieri et al., 2013). It is therefore reasonable to assume that the mammalian SFMBT2 will act like other PcG proteins and be involved in transcriptional repression. This question was addressed by performing RNA-seq analysis of *Sfmbt2*−/− extraembryonic tissues (E7.5 ectoplacental cone and extraembryonic ectoderm). When compared with wild-type littermates, the mutant tissues displayed significant upregulation of 704 genes; in contrast, only 317 genes displayed downregulation in mutant tissues (Fig. 1). GO analysis suggests most genes affected by knockout of SFMBT2 have roles in developmental processes, cell organization and biogenesis, stress response, cell cycle and proliferation, and signal transduction. Only 20 of 704 genes (0.03%) of all significantly upregulated genes and 5 of 317 (0.02%) significantly downregulated genes have GO terminologies associated with placental development. This may in part reflect the underrepresentation of GO data associated with placenta. The stage of embryogenesis chosen, E7.5, represents a stage that precedes the most dramatic phenotypic differences between mutant and wild-type embryos, observed at E8.5 (Miri et al., 2013). At E7.5, mutant embryos are generally normal in appearance, although some may have reduced extraembryonic ectoderm. One would expect that loss of a tissue would be reflected in the transcriptome by loss of transcripts from that tissue. It is therefore interesting that so few genes display downregulation in mutant tissues.

### ChIP-seq binding of SFMBT2 displays broad peaks

In fruit flies, PcG complexes bind to regulatory sequences called Polycomb Response Elements (PREs) (Khan et al., 2014; Orsi et al., 2014; Follmer et al., 2012). No clearly definitive PREs have been identified in mammalian cells, suggesting a different mechanism of regulation operates in the larger genomes of vertebrates. In order to assess whether SFMBT2 might be involved directly in transcriptional repression, we performed chromatin immunoprecipitation followed by next-generation sequencing in TSC, using both the antibody directed at endogenous SFMBT2 protein and antibody directed against FLAG; the latter was used on cells expressing a FLAG-tagged *Sfmbt2* transgene.

**Fig. 2.**
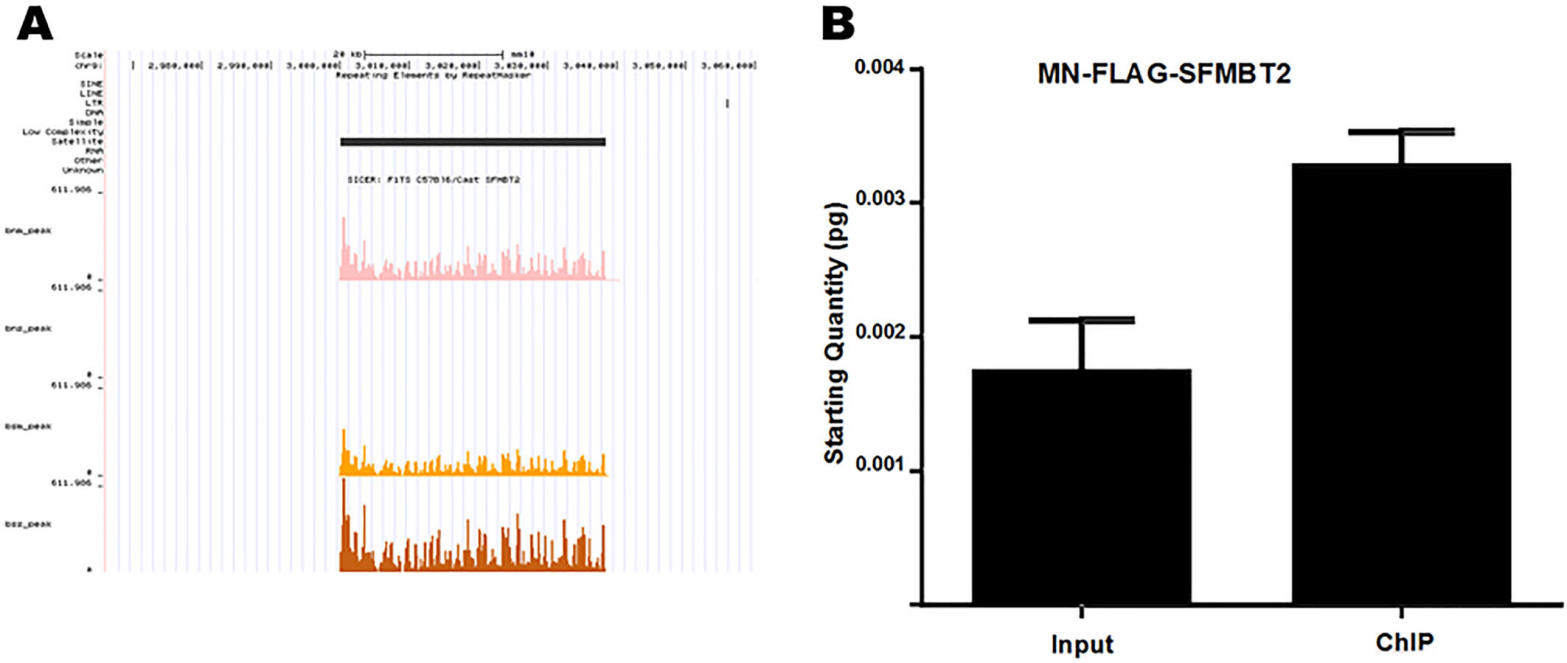
**Association of SFMBT2 with major satellite sequences.** (A) An example of a called SFMBT2 peak localized to a region enriched for major satellite sequences. The peak was called using SICER with a FDR of 0.01. Track colours are as follows: MN endogenous SFMBT2 (bnm_peak), pink; MN FLAG-SFMBT2 (bsm_peak), orange; sonicated FLAG-SFMBT2 (bss_peak), brown. Peaks for sonicated endogenous SFMBT2 (bns_peak) were not called for this region, likely due to the samples being prepared and sequenced separately. (B) Major satellite sequences were significantly enriched in ChIP DNA of MN FLAG-SFMBT2 samples relative to wild type when equal amounts of DNA were used in qPCR (*P*<0.005).

Initial data analysis with MACS2 did not generate any obvious discrete binding of SFMBT2 to the genome. Given that previous studies showed an interaction between SFMBT2 and modified histones, we subjected the data to analysis with SICER and found that SFMBT2 binds to the genome in a manner similar to that observed for histones, i.e. in modified and broad peaks. Many of these peaks map to regions rich in repetitive elements, in particular LINE sequences (Fig. 2A). Validation of association with LINE elements was performed by qPCR (Fig. 2B). No obvious peaks were located close to any of the de-repressed genes in mutant extraembryonic tissues.

### SFMBT2 associates with centromeric major satellite sequences and other repetitive elements

**Fig. 3.**
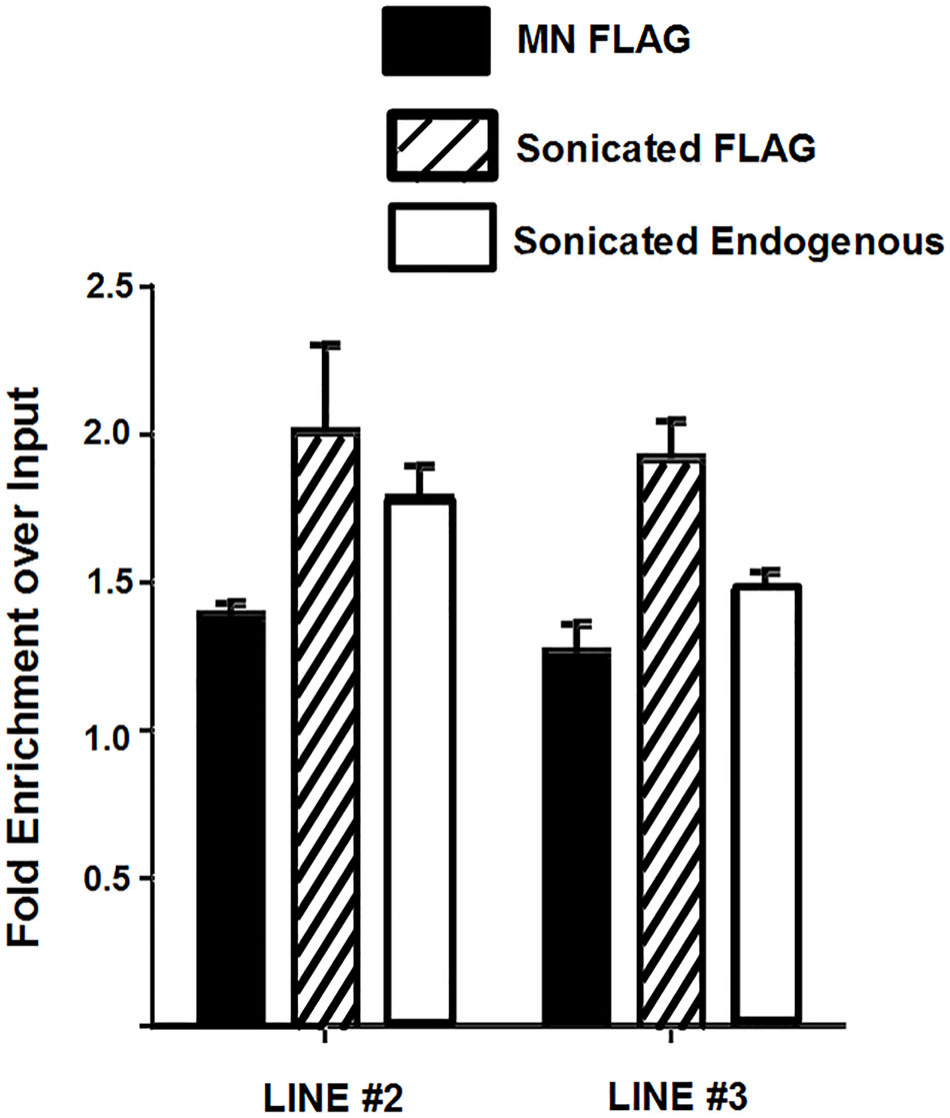
**Association of SFMBT2 with LINE elements.** Endogenous and FLAG-SFMBT2 ChIP exhibit enrichment of LINE elements relative to input approximately 1.8- to 2.0-fold even when two different primers were used. Sonicated samples likely display greater fold enrichment due to larger DNA fragments.

Immunohistochemistry in TSC clearly showed an association of SFMBT2 with centromeric regions (Fig. S1B). Because of the repetitive nature of centromeres in mammalian genomes, mapping is problematic. However, during the quality control step in which adapter sequences are removed, we noted that a significant enrichment of major satellite sequences was found in the non-adapter overrepresented sequence files in the MN-digested samples of both endogenous and FLAG immunoprecipitates (Table S2). Enrichment was confirmed by qPCR using published primer sequences (Fig. 3).

**Fig. 4.**
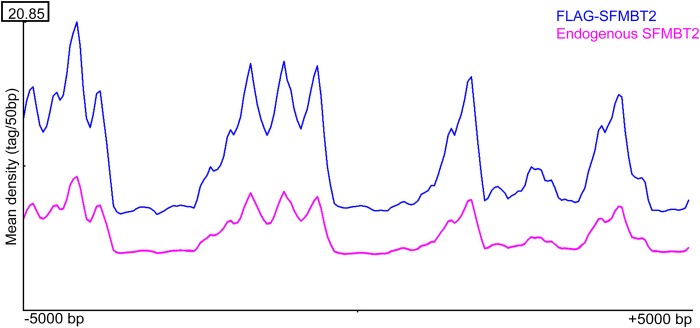
**Read distribution of SFMBT2 peaks at LINE elements across the genome.** RegionR analysis revealed similar patterns of endogenous (pink) and FLAG-SFMBT2 (blue) read distributions are observed across known LINE genomic coordinates from 5000 bp upstream to 5000 bp downstream of the centre of the specified LINE coordinates.

**Fig. 5.**
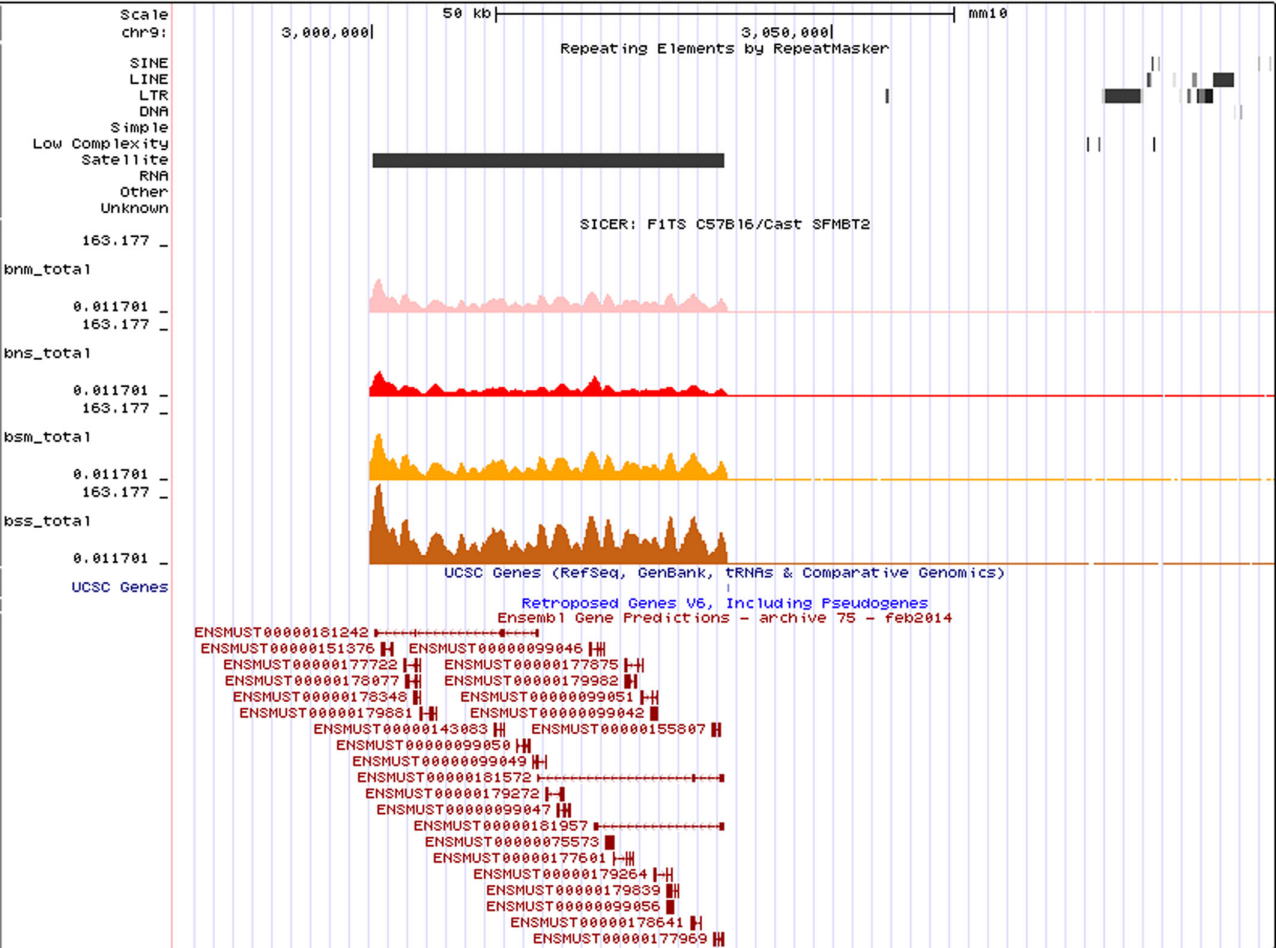
**Association of SFMBT2 with ncRNAs.** SFMBT2 peaks are mapped to a pericentromeric region enriched for major satellite sequences, which also encode for a large cluster of lncRNAs.

Binding of SFMBT2 to LINE elements and major satellite sequences prompted us to look at other classes of repetitive DNA. We used regioneR to visualize the distribution of SFMBT2 in relation to: LINE elements (Fig. 4), low complexity DNA, LTRs, ncRNA, satellite sequences, simple repeats and SINE elements (Fig. S2). Statistically significant patterns were generated for each of these classes of repetitive DNA sequence. Interestingly, association with ncRNAs was quite common (Fig. 5). A statistical analysis of broad peaks using bedtools revealed that between approximately 26% and 46% of SFMBT2 peaks associated with lncRNAs (Table 1), many of which were pseudogenes embedded in regions rich in other repetitive elements, making the design of appropriate qRCR primers problematical.

**Table 1.**
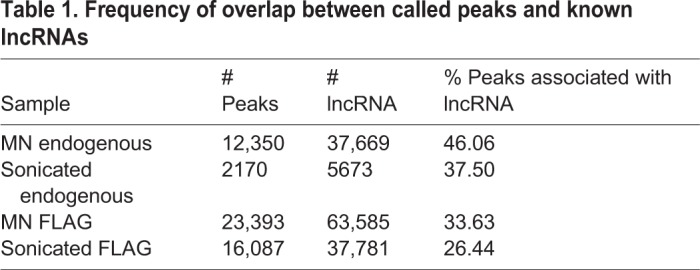
**Frequency of overlap between called peaks and known lncRNAs**

**Fig. 6.**
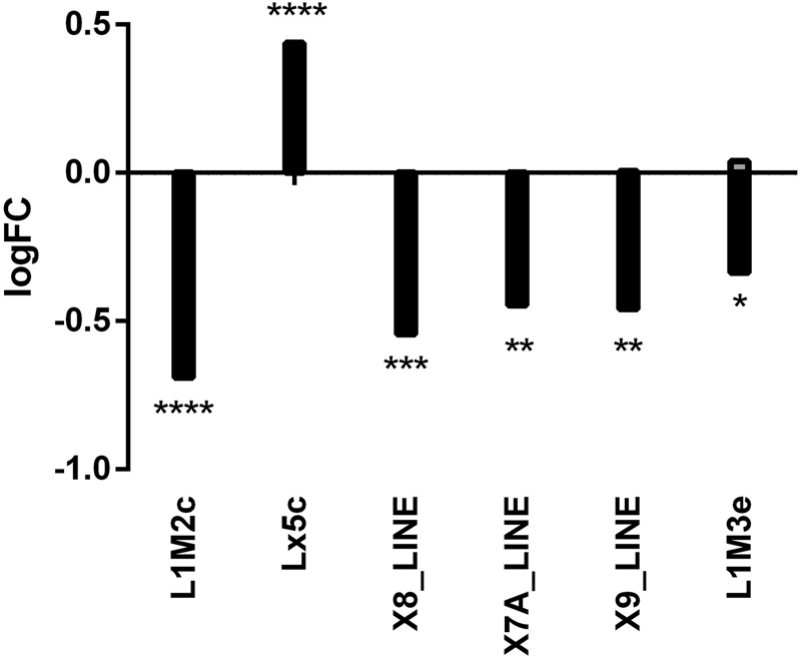
**Altered expression of LINE elements in *Sfmbt2* null tissue.** RNA-seq data from *Sfmbt2* null versus wild-type E7.5 extraembryonic tissues were subjected to RepEnrich2 analysis to assess the expression of LINE elements. Six elements were found to be altered in mutant tissues. (****FDR≤E-06; ***FDR≤0.0003; **FDR≤0.005; *FDR≤0.05).

The observation of SFMBT2 peaks at repetitive elements prompted us to look at expression of LINE sequences in *Sfmbt2* null extraembryonic tissues. Interestingly, only six displayed statistically significant (modest) changes in mutant tissues, and of those five were reduced (Fig. 6; Table S3). We can't say whether this reflects loss of regulation (positive or negative), or loss of cell types; while *Sfmbt2* null embryos at E7.5 are largely normal in appearance, it is not unusual to find some with reduced extraembryonic ectoderm.

### SFMBT2 distribution linked to stereotypical histone marks

**Fig. 7.**
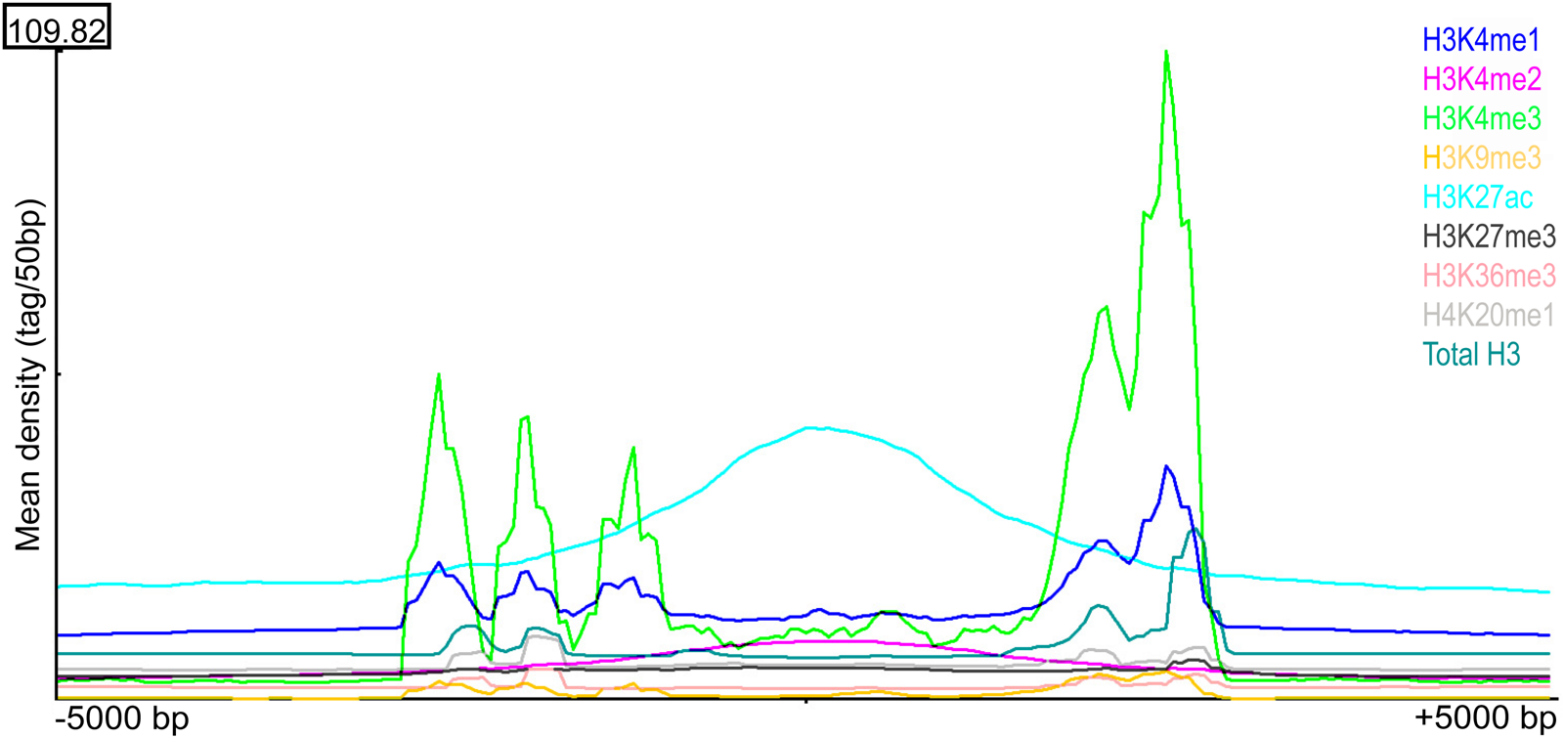
**Histone variant association with SFMBT2 peaks.** Histones exhibit three distinct patterns of distribution across called endogenous SFMBT2 peak coordinates. The distribution of ChIP-seq reads associated with each histone modification was visualized from 5000 bp upstream to 5000 bp downstream of the centre of called SFMBT2 peaks.

**Fig. 8.**
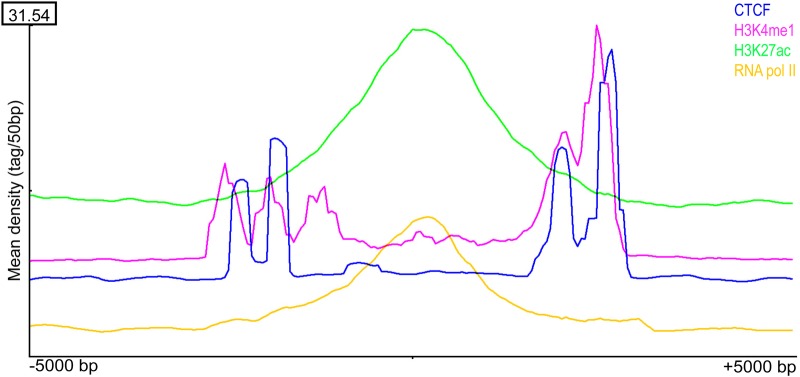
**CTCF** **association with SFMBT2** **peaks.** CTCF and H3K4me1 display different distributions across called endogenous SFMBT2 peak coordinates compared to H3K27ac and RNA pol II. The distribution of ChIP-seq reads associated with each histone modification was visualized from −5000 bp to +5000 bp of the centre of called SFMBT2 peaks.

The association of SFMBT2 with DNA features such as repetitive and major satellite sequences prompted us to examine the relationship with other histone marks in TSC. Publicly available ChIPseq data for several histone variants were mapped using SEQminer onto the SICER-defined SFMBT2 peaks. Histone H3 displayed a stereotypical distribution surrounding the middle of SFMBT2 peaks, with three strong peaks on one side within about 2.5 kb, and a fourth peak on the other side at about the same distance (Fig. 7). The strongest signal is seen in the H3K4me3 dataset. A similar pattern is observed in the CTCF dataset, while both RNA polymerase II (PolII) and H3K27ac appear to pile up over the SFMBT2 peaks (Fig. 8). These observations are unexpected, given that H3K4Me3, H3K27ac and PolII are generally associated with transcriptionally active chromatin, while SFMBT2 appears at least superficially to be transcriptionally repressive (see RNAseq section). Histone H2A also displays a stereotypical arrangement close to SFMBT2 peaks (Fig. S3).

## DISCUSSION

Progenitor cells allow a tissue to bulk up during development or repair itself following injury. Mammals are particularly dependent on stem/progenitor cells during development because embryogenesis is accompanied by an increase in mass, unlike, for example, insect or amphibian embryos. The variable size of different mammalian species may reflect, at least in part, the renewal capacity of their stem/progenitor cell populations.

The placenta, a highly specialized organ, is dependent, at least in rodents, on the integrity of the trophoblast progenitor population. Reduced numbers of TSC in embryos leads to a reduced placenta and embryonic death. Establishment of TSC has been studied extensively (Ohinata and Tsukiyama, 2014; Rossant and Cross, 2001); however, maintenance of TSC is less well understood. SFMBT2, a PcG protein, is required to maintain the TS compartment.

*Sfmbt2* mutant embryos establish a TS cell compartment, as measured by CDX2 positive cells, but the cell numbers are reduced and placenta growth does not proceed past E8.5 (Miri et al., 2013). Its loss results in de-repression of a suite of genes and premature differentiation of existing TSC into a small placenta. One of the defining features of trophoblast cells is their unusual mode of cell growth following differentiation via a process called endoreduplication, generating several different classes of trophoblast giant cells (TGC) (Simmons et al., 2007). Endoreduplication is characterized by DNA replication in the absence of mitosis, which seemingly makes the requirement for functional centromeres moot. Indeed, we have shown that some TGC in embryos completely lack any SFMBT2 at chromocentres (Miri et al., 2013), and the distribution of SFMBT2 protein in differentiated cells distal to the pool of stem cells at the base of the labyrinth becomes diffuse, further evidence that SFMBT2 function is tied to stemness in trophoblasts.

**Fig. 9.**
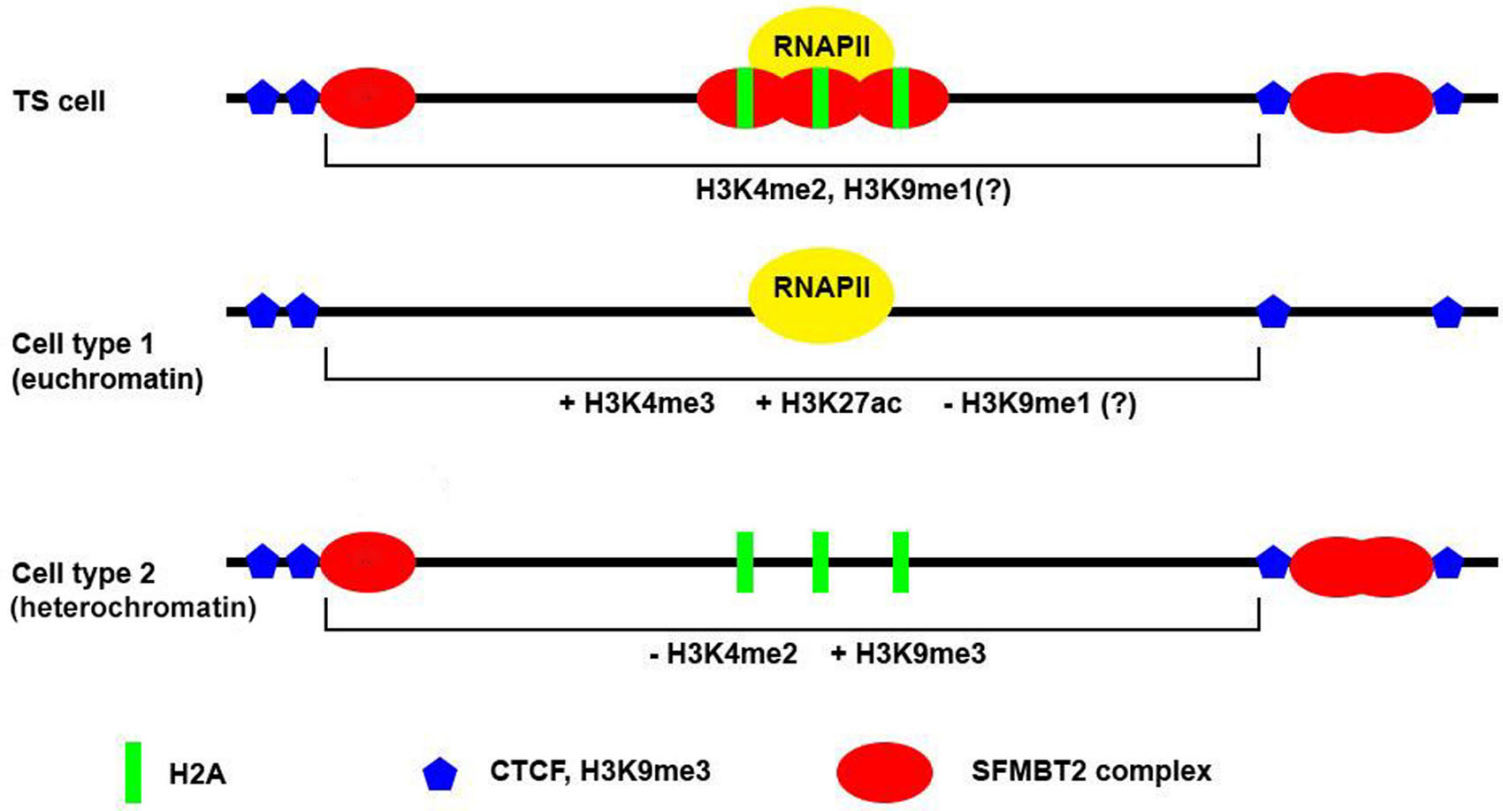
**Model explaining SFMBT2 regulation of differentiation.** SFMBT2 protein (red ovals) resides close to both activating (H3K27ac; PolII) and repressive (CTCF, blue hexagons) chromatin marks, with strong associations with all three methylated forms of H3K4, and modest association with H3K9me3. In addition, total H2A (green rectangles) is found to be centred on SFMBT2 peaks. These suggest that SFMBT2 may help define a trophoblast-specific poised state, which devolves into variable differentiation pathways when SFMBT2 is removed.

Classic PcG protein complexes in fruit flies regulate target genes by binding to discrete sequences, PREs, followed by establishment of repressed chromatin. Although a number of genes are de-repressed in *Sfmbt2* mutant extraembryonic tissues, most are upregulated by approximately twofold, and none has an SFMBT2 peak in the near vicinity; the closest peak is several Megabases (Mb) away. These data support the notion that in mammals, SFMBT2-dependent repression is secondary to the main function, and that the de-repression we observe reflects premature differentiation of extraembryonic tissues. How then is SFMBT2 maintaining the pool of undifferentiated TSC? The distribution of SFMBT2 in TSC is closely associated with features known to be involved in heterochromatin, such as LINE elements, lncRNAs and major satellite sequences. Of note, LINE1 elements have previously been shown to be essential for proper developmental progression and the self-renewal of ESCs in pre-implantation embryos through its role in transcriptional regulation (Percharde et al., 2018). While SFMBT2 may be involved in the repression of these LINE elements in TSCs to oppose an ESC phenotype (Nosi et al., 2017; Roberts and Fisher, 2011), our observation that of the small number of LINE elements with altered expression in mutant tissues, five of six display downregulation rather than upregulation, suggests they may be doing something else in trophoblast cells. The localization of SFMBT2 at pericentromeric regions in mitotic cells and chromocentres in interphase cells suggests a strong interaction with heterochromatic elements. At the same time, the stereotypical pile-up of active chromatin marks such as H3K4me3 and H3K27ac to regions flanking SFMBT2 peaks suggests that in TSC, SFMBT2 may be part of the architectural components that maintain a poised chromatin state, characteristic of other stem/progenitor cells but specific in mechanism to trophoblast, and that reduction of SFMBT2 may allow opening up of chromatin during the differentiation process (Fig. 9). The stereotypical association of CTCF with SFMBT2 peaks supports this view, given the role this boundary-element protein plays in the maintenance of stem cell integrity. Phenotypic and biochemical analysis of SFMBT2-interacting proteins in trophoblast stem cells may provide further insight into the mechanism by which this chromatin protein regulates placenta development.

The strongest association, as measured by visualization, is with chromocentres. Our ChIP-seq data contain elements from pericentromeric DNA, e.g. major satellite sequences, and while independent validation using qPCR was successful, it was modest at best, probably a reflection of the technical problems associated with analysing highly repetitive DNA. Indeed, centromeres are one of the last frontiers in genome analysis (Jain et al., 2018), their mysteries camouflaged by their repeats. ChIP-seq datasets typically exclude these regions because they are unmappable. This makes pursuit of a structural role in TS cell centromere function highly challenging. Advances in technologies aimed at studying the architectural organization of genomes in undifferentiated, differentiated and abnormal cells has revealed that large distances in the genome are bridged by loops that are defined by Topological Associating Domains (TADs) (Pombo and Dillon, 2015; Dekker and Misteli, 2015; Dixon et al., 2012). These features emerge from analysis of multiple chromatin marks in whole genome datasets. Trophoblast stem cells are currently poorly represented in the ENCODE database. The addition of SFMBT2 distribution in trophoblast stem cells reported here will build on a small but growing set of parameters that will allow more comprehensive analysis of this lineage.

## MATERIALS AND METHODS

### FLAG-tagged TSC

The lentiviral construct expressing myc-tagged *Sfmbt2* with a linked GFP transgene (Miri et al., 2013) was modified by substitution of the myc-tag with a triple FLAG tag, following digestion of the lentiviral plasmid with BstBI and XbaI, and ligation of a FLAG containing oligonucleotide with engineered sticky ends. Lentiviruses were produced as described (Miri et al., 2013) and used to infect (*C57BL6×Castaneus*) F1 TSC at early passage, kindly supplied by Dr Terry Magnuson (Department of Genetics, University of North Carolina at Chapel Hill, USA).

GFP-expressing cells were sorted by fluorescence-activated cell sorting (FACS) for three different levels of GFP: strong, medium and weak. The resulting cell pools were expanded into ‘bulk’ cell lines. The experiments described in this paper were generated with the strongly expressing TS cell line, designated 7H2-1. Immunohistochemistry revealed that FLAG distribution was indistinguishable from endogenous SFMBT2 (Fig. S1A). Following fixation in neutral buffered 10% formalin (Sigma-Aldrich, #HT501320) for 15 min, cells were rinsed with phosphate buffered saline (PBS) and permeabilized in 0.3% Triton-X for 15 min. Blocking was done in 10% goat serum and 0.1% Triton-X in PBS for 1 h at room temperature. Antibodies were diluted in antibody dilution buffer consisting of 5% goat serum and 0.1% Triton-X in PBS. Washes were performed in PBS and 0.05% Tween20. Counter staining was done using 4′,6-diamidino-2-phenylindole (DAPI) at a concentration of 0.1 µg/ml in PBS. Human anti-CREST antibody was purchased from Antibodies Incorporated (#15-234); anti-FLAG antibody was purchased from Sigma-Aldrich (M2, #F3165); anti-SFMBT2 antibody was produced in-house (Miri et al., 2013).

### Chromatin immunoprecipitation

#### Harvesting TSC

A minimum of 10^7^ cells are required for a standard ChIP experiment coupled with next-generation sequencing (ChIP-seq). TSC form colonies in culture which minimizes cellular differentiation. However, an accurate cell count requires the dissociation of TSC, which may result in stress-induced differentiation or cell death. To circumvent this problem, we calculated the weight of 10^7^ cells, and used this value as a proxy for cell counting. Cells were trypsinized twice with a recovery period of 1 day between trypsin treatments. TSC were washed with PBS and incubated with 0.25% trypsin at 37°C for 2 min. Four volumes of standard media were added to neutralize trypsin function and the solution was agitated through pipetting up and down to break cell–cell interactions. Cells were pelleted and resuspended in TS media, plated and incubated overnight to minimize cellular stress. The trypsin treatment was repeated using a pre-weighed tube and the cell resuspension was used for cell counting by hemocytometer. The remaining cells were pelleted and weighed. 10^7^ TSC weigh ∼0.23 g.

#### Cross-linking with formaldehyde

Plates of TSC were washed three times with cold PBS and fixed with 1% formaldehyde at room temperature for 10 min. Formaldehyde was quenched through addition of glycine to a final concentration of 0.125 M at room temperature for 5 min. Cross-linked cells were washed three times with cold PBS and harvested in cold PBS supplemented with phenylmethylsulfonyl fluoride (PMSF) and protease inhibitors using a rubber cell scraper. Harvested cells were then aliquoted into pre-weighed tubes for a final weight of 0.23 g. Cells were flash-frozen using a dry ice-ethanol slurry and stored at −80°C until use.

#### Chromatin immunoprecipitation by sonication (SChIP)

Frozen TSC pellets were thawed on ice for 30 min and chromatin precipitation was performed as previously described by Rada-Iglesias et al. (2011). Briefly, chromatin was sonicated to an average size of 75 bp–400 bp. Sonication parameters were as follows: total processing time of 5 min, amplitude of 40, pulse duration of 10 s and cooling duration of 30 s. Sonicated chromatin was incubated overnight at 4°C with a 5 µl aliquot of anti-SFMBT2 antibody (Miri et al., 2013), then with 100 µl protein G Dynabeads (Thermo Fisher Scientific, #10009D) for immunoprecipitation of endogenous SFMBT2 for 2 h at 4°C, or with 40 µl FLAG-conjugated beads for immunoprecipitation of FLAG-SFMBT2 (Sigma-Aldrich, #M8823). Beads were washed five times with cold RIPA buffer and once with cold TE buffer supplemented with 50 mM NaCl. After phenol-chloroform extraction, aqueous fractions were heated at 55°C for 5 min to remove residual phenol-chloroform then cooled to room temperature for 2 min prior to an ethanol-based DNA precipitation. Ethanol-washed DNA pellets were heated at 37°C for 5 min to remove residual ethanol. Samples were then incubated with nuclease-free water at room temperature for 2 min and vortexed. Resuspended DNA was quantified by PicoGreen and stored at −20°C.

#### Chromatin immunoprecipitation by micrococcal nuclease digestion (MN ChIP)

Cells were thawed on ice for 30 min and chromatin immunoprecipitation was performed as previously described (Tsankov et al., 2015) until the wash procedure. Pellets were resuspended in 1 ml lysis buffer and incubated on ice for 1 h. Chromatin fragmentation was achieved using 418 units of micrococcal nuclease (MN; Worthington Biochemical Corporation, #LS004797) with incubation at 37°C for 2.5 h. Digestion was inhibited by addition of EGTA until a final concentration of 50 mM. Washes, elution and DNA purification steps were performed as in SChIP.

#### Library preparation and high-throughput sequencing

ChIP and total input DNA was sent to The Centre for Applied Genomics (TCAG) for library preparation and subsequent next-generation sequencing. Equal amounts (10 ng) of input and ChIP DNA of sonicated endogenous SFMBT2 samples was supplied for library preparation using the Illumina TruSeq protocol. Sonicated FLAG-SFMBT2 and all MN ChIP samples were prepared using the NEB Ultra DNA library preparation protocol. The NEB Ultra DNA library preparation protocol was used because of low ChIP DNA yields. Although all libraries were subject to paired-end sequencing on the Illumina HiSeq 2500 platform, sonicated endogenous SFMBT2 libraries were sequenced at a different time.

### RNA-seq

*Sfmbt2* null embryos were generated by inter-crossing heterozygous males and females from the *Sfmbt2* gene trap (gt) colony (Miri et al., 2013). Embryos at E7.5 of development were individually dissected into either embryo (DNA extraction buffer – genotyping) or extraembryonic (RNA extraction buffer) portions. Following genotype identification, homozygous mutant or wild-type RNA extracts from 10–20 embryos were pooled and processed for RNA-seq by TCAG using the Illumina TruSeq protocol and paired end next-generation sequencing on the Ilumina HiSeq 2500 platform. Two biological replicates of each were analyzed. Animals used in this study were housed in the Biological Sciences Facility of the Faculty of Arts and Sciences at the University of Toronto, and procedures were approved by the Local Animal Care Committee that oversees protocol design to ensure compliance with standards set out by the Canadian Council on Animal Care.

### Bioinformatics ChIPseq

Illumina fastq data files acquired from sequencing by TCAG necessitated the use of computational programs for data analysis due to the dense information content. All bioinformatics programs were run using an Ubuntu 14.04 OS.

#### Quality assessment and adaptor trimming

The wrapper tool TrimGalore was used to facilitate quality assessment and adaptor trimming (Krueger, 2015). FastQC was used for assessing the quality of sequencing reads, identification of adaptor sequences, and identification of non-adaptor overrepresented sequences (Andrews, 2010). Adaptor sequences of paired-end reads were trimmed using CutAdapt (Martin, 2011). Quality of trimmed reads was then reassessed to ensure removal of adaptor sequences.

#### Genome alignment and peak calling

Adaptor-trimmed sequences were aligned to the mm10 *Mus musculus C57Bl6* genome annotation via Bowtie2 with the quality-check filter implemented (Langmead and Salzberg, 2012). Narrow peaks were called using the MACS2 program with a false discovery rate (FDR) of 0.01 (Zhang et al., 2008). Broad peaks were called using SICER v1.1 with a FDR of 0.01, a window of 200 bp, and a gap of 1000 bp (Zang et al., 2009). Prior to invoking SICER, modifications were made to the GenomeData.py code for inclusion of the mm10 genome. A statistical comparison of genome-wide count distributions was performed using the University of California Santa Cruz (UCSC) utility wigCorrelate (Table S1) (Jee et al., 2011). Due to high correlation between replicates, replicates were pooled and subject to peak calling. All subsequent analyses were performed using SICER-called peak sets.

#### Assessing peak conservation across samples

The R package DiffBind was used for comparison of called peaks across samples (Stark and Brown, 2011). DiffBind enabled depiction of similarity between called peaks in a heatmap and generated a consensus peak set. Venn diagrams were generated for visualization of peak conservation in the consensus peak set for each pooled sample and conserved peaks between samples. The fraction of total reads found in called peaks (FRiP) was also calculated.

#### Statistical assessment of SFMBT2 peak association with genomic features

The R package regioneR was used to assess the association between SFMBT2 peak sets with genomic repeats and ncRNA (Gel et al., 2016). Genomic coordinates for mm10 repeats were acquired from RepeatMasker through the UCSC table browser function. Coordinates in the genomic repeat file were binned according their respective repeat families. Information in each repeat family bin was then formatted into BED files for use in regioneR. The same approach was used for ncRNA coordinates acquired from the NONCODE website (Bu et al., 2012). 1000 permutation tests were run per sample with a seed number of 1.

#### Assessing distribution of available ChIP-seq reads across supplied coordinates

Publicly available ChIP-seq data in TSC were downloaded from NCBI GEO. The associated accession numbers are: CTCF (GSM998993), RNA polymerase II (GSM967644), H3K4me1 (GSM1035385), H3K4me2 (GSM967645), H3K4me3 (GSM1035382), H3K27ac (GSM967654), H3K27me3 (GSM967649), H3K36me3 (GSM967646), Total H3 (GSM967647), and Total H2A (GSM1015786, GSM1015787, GSM1015788), H3K9me3 (GSM1035383), H4K20Me1 (GSM967655). All available mm9 BED files were converted to mm10 through the UCSC liftOver utility with automated processing of headers in command-line. NCBI accession numbers with no available BED files were processed from SRA files. SRA files were processed using the sratoolkit to generate fastq files which were subsequently mapped to the mm10 genome using Bowtie2.

SeqMiner was used for read pile-up assessments across specified genomic coordinates (Ye et al., 2014). The shell command for invoking SeqMiner was modified from ‘java – Xmx2000 m – jar seqMINER.jar’ to ‘java –Xmx15000 m – jar seqMINER.jar’ for increased memory usage. xGenomic coordinates of called SFMBT2 peaks or repetitive elements in BED format were supplied as a reference file. Aligned read files for histone or protein distributions of interest were in BED or BAM format. A seed value of 1 was used for all analyses and analysis window was ±5000 bp from the centre of coordinates supplied in the reference file.

### Bioinformatics RNAseq

Fastq files acquired from TCAG were analyzed according to the protocol described by Trapnell et al. (2012). Reads were aligned to the C57Bl6 mm10 genome using TopHat2 and subsequent analysis was performed using the Tuxedo suite (i.e. Cufflinks, Cuffmerge, Cuffdiff and the R package CummeRbund). Data were further analyzed using SeqMonk. General Gene Ontology (GO) analysis was performed using the GO analysis tool at Mouse Genome Informatics (MGI) website (www.informatics.jax.org). Significantly differentially expressed genes identified by CuffDiff were cross-analyzed with a list of genes associated with placenta-specific GO terminologies acquired from EMBL-EBI's QuickGO (www.ebi.ac.uk/QuickGO/).

### RepEnrich2 analysis for differential expression of LINE elements in RNA-seq

An updated method of RepEnrich (i.e. RepEnrich2) as detailed by Criscione et al. (2014) was applied for the analysis (https://github.com/nerettilab/RepEnrich2). Of note, all available python codes were converted from python 2.7 to python 3.0 for use. To reiterate, a bed file detailing the mm10 coordinates of known repetitive LINE elements was extracted from the UCSC genome browser. RNA-seq data of replicate *Sfmbt2* null and wild-type extraembryonic tissues were mapped to the mm10 reference genome. RepEnrich2_subset.py was executed to parse aligned sequences into uniquely and multi-mapping files. RepEnrich2.py was then applied using the custom mm10 LINE bed file for all samples. Differential enrichment analysis of the RepEnrich2 output was performed using the R package ‘edgeR’.

### Validation ChIP qPCR

Standard qPCR was performed using 20 pg of sonicated FLAG-SFMBT2 template whereas 50 pg of MN-digested FLAG-SFMBT2 template was used. Standards used consisted of serial dilutions of their respective total input fractions. qPCR was performed using the WISENT advanced qPCR mastermix with supergreen lo-rox reagent (WISENT Bioproducts, #800-435-UL). Annealing temperatures for all qPCRs was set at 57°C with 40 amplification cycles.

#### Primer design

Common peaks called across pooled ChIP samples were identified as potential targets. Genomic sequences were then subject to primer design using the Primer3 program. Potential amplicons corresponding to each putative primer set were identified by Primer-BLAST. Primer pairs which generate a single amplicon within 1000 bp were then tested by end-point PCR using input template. Only primers that gave rise to a single distinct amplicon were used for qPCR. Negative targets were designed in the same manner for regions where no fold enrichment over input was observed.

#### Major satellite qPCR

Primers used for major satellite qPCR were previously published by Martens et al. (2005). qPCR could only be performed on MN-digested FLAG-SFMBT2 samples; 0.5 pg of template was amplified with 1 µM primer.

## Supplementary Material

Supplementary information
