# Supplementary material for: Unique trophoblast chromatin environment mediated by the PcG protein SFMBT2
Source: Biol Open. 2019 Aug 7;8(8):bio043638. doi: 10.1242/bio.043638 (PMC6737984; doi:10.1242/bio.043638)
Supplement: Supplementary information [file biolopen-8-043638-s1.pdf]

Supplementary Data

Table S1. Pearson's correlation (wigCorrelate) scores reflecting read distribution similarity between samples.

**Correlation between *C57Bl6* and *Castaneus***		
**Sample 1 (*C57Bl6*)**	**Sample 2 (*Castaneus*)**	**wigCorrelate score**
MN endogenous	MN endogenous	0.90243
Sonicated endogenous	Sonicated endogenous	0.91838
MN FLAG	MN FLAG	0.90723
Sonicated FLAG	Sonicated FLAG	0.90834
**Correlation between endogenous and FLAG-SFMBT2**		
Sample 1 (endogenous)	Sample 2 (FLAG)	wigCorrelate score
MN (C57Bl6)	MN (C57Bl6)	0.991147
Sonicated (C57Bl6)	Sonicated (C57Bl6)	0.885683
MN (Castaneus)	MN (Castaneus)	0.989347
Sonicated (Castaneus)	Sonicated (Castaneus)	0.904497

**Correlation between replicates (C57Bl6; no FDR)**		
**Sample 1**	**Sample 2**	**wigCorrelate score**
MN endogenous 1 (BNM1)	MN endogenous 2 (BNM2)	0.986783
Sonicated endogenous 1 (BNS1)	Sonicated endogenous 2 (BNS2)	0.941913
MN FLAG 1 (BSM1)	MN FLAG 2 (BSM2)	0.969138
Sonicated FLAG 1 (BSS1)	Sonicated FLAG 2 (BSS2)	0.987105

**Correlation between replicates (C57Bl6; FDR1E-2)**		
**Sample 1**	**Sample 2**	**wigCorrelate score**
MN endogenous 1 (BNM1)	MN endogenous 2 (BNM2)	0.986783
Sonicated endogenous 1 (BNS1)	Sonicated endogenous 2 (BNS2)	0.950948
MN FLAG 1 (BSM1)	MN FLAG 2 (BSM2)	0.990476
Sonicated FLAG 1 (BSS1)	Sonicated FLAG 2 (BSS2)	0.998661

Biology Open • Supplementary information

Table S2. All non-adaptor over-represented sequence frequencies as identified by FastQC

Sequence	Sample	% of reads
ACGTGAAAAATGAGAAATGCACACTGAAGGACCTGGAATATGGCGAGAAA	MN_FLAG_2R1	0.103
CGTCATTTTTCAAGTCGTCAAGTGGATGTTTCTCATTTTCCATGATTTTC	MN_FLAG_2R1	0.113
TCCACCTTTTTCAGTTTTCCTCGCCATATTTCACGTCCTAAAGTGTGTAT	MN_endogenous_1R1	0.106
	MN_endogenous_2R1	0.127
	MN_endogenous_2R2	0.117
	MN_FLAG_1R1	0.128
	MN_FLAG_1R2	0.121
	MN_FLAG_2R1	0.165
	MN_FLAG_2R2	0.158
TCCACGTCCTACAGTGGACATTTCTAAATTTTCCACCTTTTTCAGTTTTC	MN_FLAG_2R1	0.119
	MN_FLAG_2R2	0.101
TCCGTGATTTTCAGTTTTCTCGCCATATTCCAGGTCCTTCAGTGTGCATT	MN_endogenous_2R1	0.112
	MN_endogenous_2R2	0.102
	MN_FLAG_1R1	0.111
	MN_FLAG_1R2	0.102
	MN_FLAG_2R1	0.160
	MN_FLAG_2R2	0.148
TCGTCATTTTTCAAGTCGTCAAGTGGATGTTTCTCATTTTCCATGATTTT	MN_FLAG_2R1	0.120
	MN_FLAG_2R2	0.106
TCTCATTTTCCGTGATTTTCAGTTTTCTCGCCATATTCCAGGTCCTTCAG	MN_FLAG_2R1	0.127
	MN_FLAG_2R2	0.126
TGAGAAACATCCACTTGACGACTTGAAAAATGACGAAATCACTAAAAAAC	MN_FLAG_1R1	0.101
	MN_FLAG_2R1	0.143
	MN_FLAG_2R2	0.123
TGTCCACTGTAGGACGTGGAATATGGCAAGAAAACTGAAAATCATGGAAA	MN_FLAG_2R1	0.118

Table S3. Altered Expression of LINE elements in *Sfmbt2* null extraembryonic tissues (see attached excel file).

Click here to Download Table S3

Biology Open • Supplementary information

Figure S1 Immunohistochemistry Showing Co-localization of Endogenous SFMBT2 with FLAG-tagged SFMBT2 (A) and with CREST (B). White bar represent 10 μm.

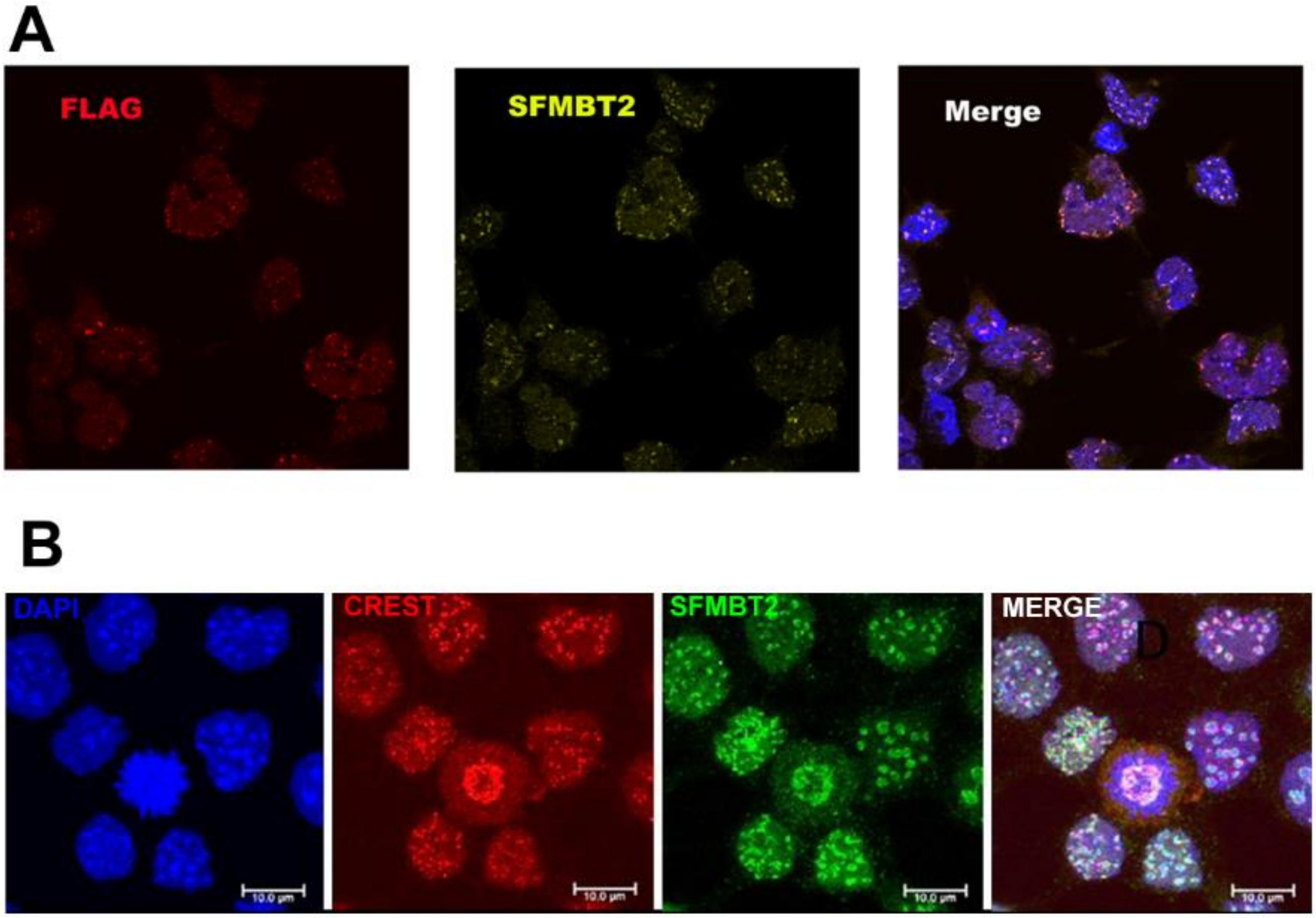

Biology Open • Supplementary information

Biology Open (2019): doi:10.1242/bio.043638: Supplementary information

Figure S2. RegionR Distribution of SFMBT2 peaks in relation to different classes of repetitive sequence. Blue lines represent FLAG –SFMBT2 peaks; pink lines represent endogenous SFMBT2 peaks.

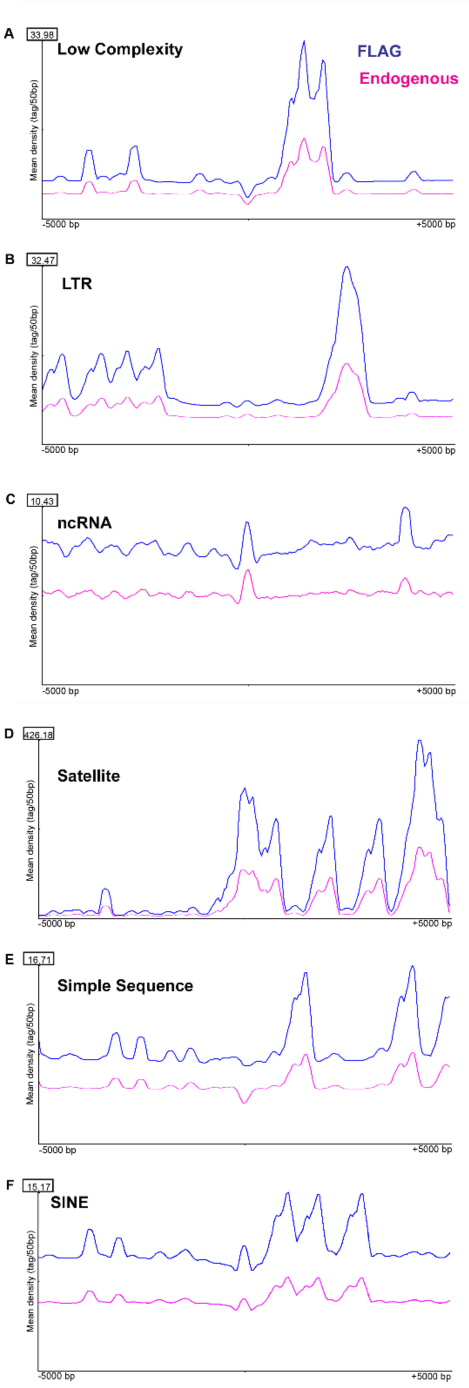

Biology Open • Supplementary information

Figure S3. Association of Total Histone H2A with SFMBT2 peaks using RegionR. ChIP-seq peaks identified using either endogenous (A) or FLAG (B) antibodies produced similar results.

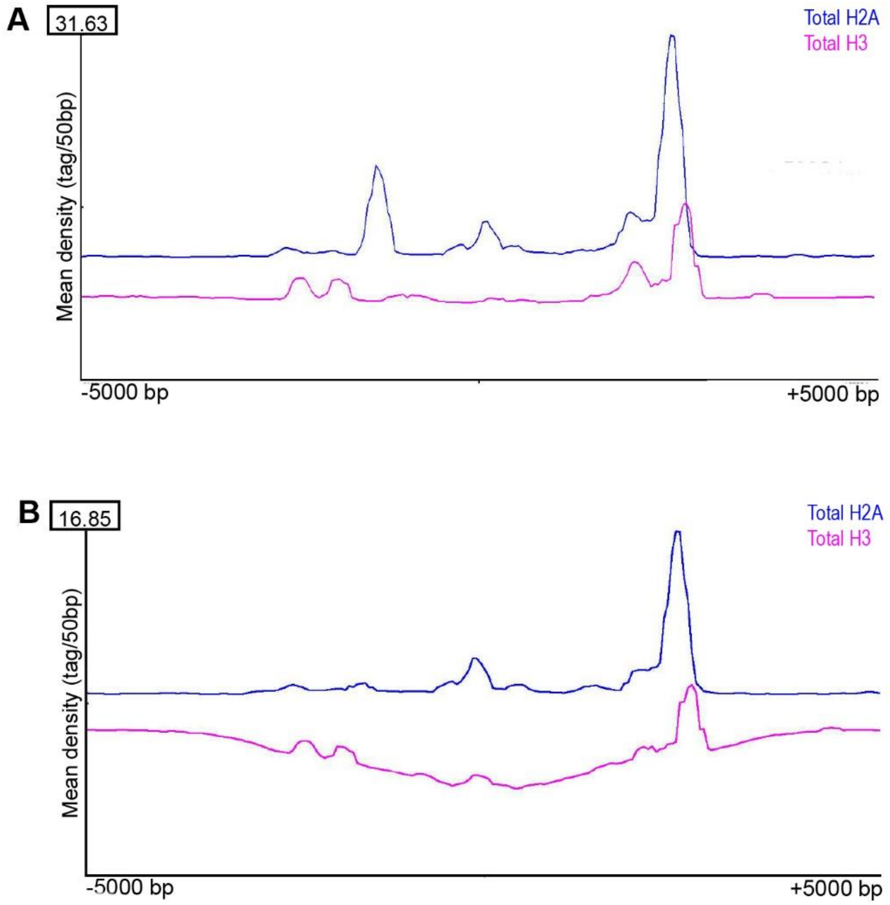

Biology Open • Supplementary information
